# Evaluation of Efficacy of ALK Inhibitors According to Body Mass Index in ALK Rearranged NSCLC Patients—A Retrospective Observational Study

**DOI:** 10.3390/cancers15133422

**Published:** 2023-06-30

**Authors:** Marco Siringo, Gabriella Gentile, Salvatore Caponnetto, Isabella Sperduti, Daniele Santini, Enrico Cortesi, Alain Jonathan Gelibter

**Affiliations:** 1 Medical Oncology Unit B, Department of Radiology, Oncology and Pathology, Policlinico Umberto I, Sapienza University of Rome, 00161 Rome, Italy; 2 Medical Oncology Department, Hospital Universitario 12 de Octubre, Instituto de Investigación i+12, 28041 Madrid, Spain; 3 Department of Biostatistics Unit, IRCCS—Regina Elena National Cancer Institute, 00144 Rome, Italy; 4 Medical Oncology Unit A, Department of Radiology, Oncology and Pathology, Policlinico Umberto I, Sapienza University of Rome, 00161 Rome, Italy

**Keywords:** ALK, Alectinib, NSCLC, BMI, predictive biomarker

## Abstract

**Simple Summary:**

ALK-rearranged non-small cell lung cancer treatment has radically changed in the last few years thanks to the development of ALK inhibitors, which clearly improved survival. To our knowledge, even though many studies correlate body mass index (BMI) with lung cancer immunotherapy, conflicting results have emerged in non-smoker patients treated with other drugs. Indeed, it is unclear whether body size variables and metabolism could affect ALK-inhibitor efficacy. The aim of this retrospective study is to correlate BMI status with survival outcomes in patients treated with commonly used ALK inhibitors in different lines of treatment. We observed that BMI status could impact survival outcomes, particularly in patients treated with Alectinib as the first line. Further prospective studies should examine this interesting phenomenon.

**Abstract:**

No evidence exists as to whether body mass index (BMI) impairs clinical outcomes from ALK inhibitors (ALKi) in patients with ALK-rearranged non-small cell lung cancer (NSCLC). Retrospective data of patients affected by metastatic ALK-rearranged NSCLC treated with ALKi were collected. We divided patients into “low- BMI” (≤25 kg/m^2^) and “high- BMI” (>25 kg/m^2^) categories and correlated them with overall survival (OS) and progression-free survival (PFS). We included 40 patients treated with ALKi. We observed a 3-year OS of 81.5% in high-BMI vs. 49.6% in low-BMI categories (*p* = 0.049); the 3-year first-line PFS was superior in high-BMI vs. low-BMI patients (47% vs. 19%, *p* = 0.019). As expected, patients treated with Alectinib had a 55.6% 3-year PFS vs. 7.1% for others treated with ALKi (*p* = 0.025). High-BMI was associated with a 100% 3-year PFS rate vs. 25.4% in low-BMI Alectinib patients (*p* = 0.03). BMI was independently correlated with first-line PFS and OS at multivariate analysis with PS (HR 0.39, CI 95% 0.16–0.96, *p* = 0.042; HR 0.18, CI 95% 0.05–0.61, *p* = 0.006). High-BMI was associated with higher efficacy in ALK-rearranged patients. These results are particularly exciting for Alectinib and could be correlated to mechanisms that should be investigated in subsequent prospective studies.

## 1. Introduction

Lung cancer is the leading cause of cancer-related death with a mortality rate of 18.4%. It is the first in men and second in women worldwide, representing the cancer type with the lowest five-year survival rate [1]. 

Tobacco smoking remains the leading cause, but several other factors have been described as risk factors for lung cancer, including exposure to asbestos, arsenic, radon, and polycyclic aromatic hydrocarbons unrelated to tobacco. In any case, an increased rate of NSCLC diagnoses was observed in non-smokers [2,3]. 

Accordingly, patients affected by NSCLC without the smoking relationship are considered a different entity because a large number of molecular and genetic mutations could be identified. On this basis, the oncogene-addicted NSCLC population is currently treated with targeted therapies discovered in the last few years [4].

*ALK* rearrangement is detectable in 2–7% of the lung cancer population. Notably, it is most common in specific categories of patients such as women, non-smokers or light smokers, young people, those with adenocarcinoma histology (often with a signet ring or acinar features), and the Asiatic population [5,6,7].

Moreover, 70% of the *ALK*-rearranged population develops central nervous system (CNS) metastasis, while almost 30% of patients, at the time of diagnosis, contribute significantly to the improvement of morbidity during their disease course [8]. 

Small-molecule tyrosine kinase inhibitors (TKIs) that target *ALK* have been introduced in the last 10 years, leading to improved progression-free survival (PFS), overall survival (OS), and overall response rate (ORR) in patients with ALK-rearranged NSCLC in various clinical trials [9].

The phase III Alex trial compared Alectinib (a second-generation ALK TKI) with Crizotinib (a first-generation *ALK* TKI), showing a median PFS (mPFS) of 34.8 months, an ORR of 83%, an intracranial response rate of 81%, and a 5-year OS rate of 62.5% [10,11]. 

Moreover, Brigatinib yielded similar results in the phase III ALTA 1L with mPFS of 24 months, ORR of 74%, 27.9 months of median intracranial duration of response in patients with measurable brain metastases at baseline, and a 3-year intracranial PFS rate of 31%, compared to Crizotinib [12].

Lorlatinib is a third-generation ALK inhibitor that can overcome the secondary resistance mutations that emerge after treatment with first-generation and second-generation *ALK* inhibitors, and it shows better intracranial activity than Crizotinib. A phase II trial demonstrated an ORR of 47% and 39% in patients treated with Lorlatinib and at least one *ALK* inhibitor and one or more ALK TKIs, respectively [13]. 

Recently, a phase III Crown trial compared Lorlatinib to Crizotinib in untreated patients and demonstrated a PFS at 12 months of 78% vs. 39%, ORR of 76% vs. 58%, and an intracranial response rate of 82% vs. 23%, respectively [14].

Based on these results, *ALK* TKIs have become the standard regimen for patients with advanced NSCLC harboring an *ALK* rearrangement. Alectinib, Brigatinib, and Lorlatinib are the preferred options but, in the absence of head-to-head comparisons, the optimal choice for first-line therapy in *ALK*-rearranged lung cancer remains unclear [15]. 

In recent years, obesity has become a public health problem because of high incidence all around and correlated comorbidities such as hypertension, hyperlipidemia, and hyperglycemia together, constituting metabolic syndrome (MS) [16].

Obesity has long been associated with worse cancer outcomes probably because of systemic physiologic alterations such as higher insulin resistance, chronic inflammation, and abnormal nutrient homeostasis, which may contribute to oncogenic transformations [17,18].

BMI (body mass index) has commonly been used as a surrogate indicator of MS and is calculated as weight in kilograms divided by height in meters squared. BMI association with cancer has been widely studied, showing conflicting results. Indeed, breast, ovarian, and colorectal cancer are associated with high mortality in obese patients while recent studies have shown a possible positive correlation between BMI and lung cancer survival. This phenomenon is called the “obesity paradox”. Nevertheless, poor health status associated with nutritional deficiency and consequent weight loss could contribute [19,20,21,22].

Moreover, lung cancer patients with higher BMI and receiving immune checkpoint inhibitors (ICIs) had better outcomes probably because of increased adipose tissue that could help to regulate immune homeostasis, constituting a trigger for immune response; in retrospective studies, the median time to treatment failure and PFS were significantly longer in overweight/obese patients compared with non-overweight patients in 976 patients affected by NSCLC treated with ICIs. Similarly, higher baseline BMI resulted in an increased ORR and PFS in NSCLC patients treated with Pembrolizumab as a first-line treatment (962 patients) and not with chemotherapy (426 patients) [23,24].

On the other hand, conflicting results emerged in the analysis of the correlation between BMI and outcomes in the *EGFR* mutated NSCLC population. In a real-world study with patients treated with second-generation *EGFR* TKIs, high-BMI correlated positively with first-line PFS, primarily in men. These results are not confirmed in the EGFR population treated with second-line Osimertinib, where BMI did not or even negatively influence survival outcomes [25,26,27].

Here, we present the clinical outcomes analysis of patients affected by metastatic NSCLC treated with *ALK* inhibitors according to baseline BMI, evaluated at the time of metastatic status. 

## 2. Materials and Methods

### 2.1. Study Design

We conducted a retrospective observational study at Sapienza University of Rome at Policlinico Umberto 1, Oncologia B Department to evaluate the efficacy of anti-*ALK* treatment and clinical predictive correlations.

Patients were considered eligible for this study if they had a histological or cytological diagnosis of stage IV NSCLC according to the American Joint Committee on Cancer (AJCC) and *ALK* rearrangement tested with next generation sequencing (NGS), fluorescent in situ hybridization (FISH), or immunochemistry (IHC).

Patients without at least one measurable lesion according to the Response Evaluation Criteria in Solid Tumors (RECIST) version 1.1 were excluded.

We collected patients’ data, including age at diagnosis, gender, Eastern Cooperative Oncology Group (ECOG) Performance Status (PS) at the time of first-line treatment, smoking status, clinical staging, tumor histology, presence of central nervous system (CNS) metastasis, and the total number of metastases. At the same time, we analyzed the presence of comorbidities focusing on cardiac and renal diseases and diagnosis of diabetes mellitus type 2 associated with the evaluation of concomitant medications. No analytical data were collected because they were not available in all populations at the time of diagnosis.

### 2.2. Classifications

Patients were divided according to smoking status: current smoker, former smoker (if they had smoked less than 10 pack years with smoking cessation time superior to 15 years), and never been a smoker (if they had smoked less than 100 cigarettes in their lifetime history).

Moreover, we calculated BMI status using the weight/height^2^ formula (kg/m^2^) and divided patients into two categories: BMI ≤ 5 mg/m^2^ (called “low-BMI”) and BMI > 25 kg/m^2^ (called “high-BMI”). This dual division was necessary to distinguish low–normal and overweight/obese patients at the time of metastatic status.

Patients received at least one *ALK* inhibitor between Crizotinib, Ceritinib, Alectinib, Brigatinib, and Lorlatinib, but also in some rare cases, patients received platinum-based chemotherapy or ICIs in different lines of the metastatic setting. Patients who experienced death or progression of disease before radiographic evaluation were excluded.

### 2.3. Statistical Analysis 

We reported continuous variables as median and range, and categorical variables as count and percentage. Progression-free survival (PFS) was defined as the time from treatment initiation until disease progression or last contact without progression disease; overall response rate (ORR) was defined as the portion of patients experiencing an objective response (complete or partial response) as best response to treatment; and overall survival (OS) was defined as the time between the start of first-line treatment and death for any cause or last contact. We estimated the OS and PFS with the Kaplan–Meier survival curves. The log-rank test was used to assess differences between groups. The median period of follow-up was calculated according to the reverse Kaplan–Meier method.

The correlation between ORR and other variables was examined by a binary logistic regression model. Univariate and multivariate Cox models were used to compare the clinical, biological, and pathological characteristics of PFS and OS. Median OS and PFS were reported with 95% confidence intervals (CIs). Significance was defined at *p* ≤ 0.05 level. Statistical analyses were performed using IBM SPSS Statistics version 23 (IBM, Armonk, NY, USA).

## 3. Results

### 3.1. Patients Characteristics

A total of 40 patients treated with ALKi from April 2013 to November 2022 were identified. The median follow-up was 47 months.

A summary of all clinicopathological characteristics of the study population is displayed in Table 1.

The majority of patients (62.5%) were female, and the median age at diagnosis was 63 years. 

In total, 32% of the population were PS0, 62,5% were PS1, and 5% were PS2. All patients suffered from adenocarcinoma, stage IV disease, or postoperative recurrence, according to the American Joint Committee on Cancer (AJCC). The median number of metastases was four; 80% and 50% of patients had visceral and brain metastasis, respectively, while 67% had more than three metastases. More than half of patients were non-smokers (52%), while 30% were former or light smokers. BMI was higher than 25 mg/m^2^ in 37.5%, with few patients affected by diabetes mellitus type 2 (DM2) (only seven) and, consequently, four patients received metformin. In addition, 47% of patients received steroids at diagnosis, and 40% received antiepileptic drugs.

Cardiac and renal comorbidities were detected in almost 13% of the population who were examined.

Out of all patients, 47% (7/15) were PS0 at diagnosis in the overweight/obese population, while 32% (8/25) were underweight/normal patients.

ALKi were evaluated in different metastatic lines; the most used was Alectinib, received in 23, 9, and 2 patients in first-, second-, and third-line treatment, respectively. 

In total, 57.5% and 85.5% of patients did not receive second- and third-line treatment. A small percentage of patients did not receive anti-ALK inhibitors as a first-line treatment but rather chemotherapy and immunotherapy (three and two patients, respectively) because of subsequent diagnosis of ALK rearrangement.

### 3.2. Association between BMI and Outcomes

**Figure 1:**
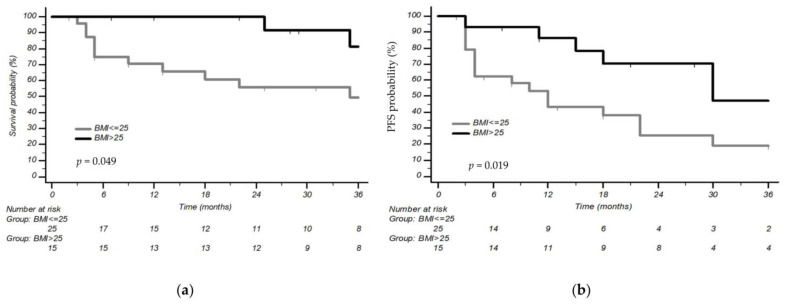
Overall survival (**a**) and progression-free survival of first-line treatment (**b**) in the overall population according to body mass index (BMI).

We grouped patients according to BMI status. We discovered an increased OS in patients with high-BMI compared with low-BMI, with a 3-year OS of 81.5% vs. 49.6%, and an mOS of 74 months (CI 95% 35–74 months) vs. 35 months (CI 95% 9–62 months), respectively (*p* = 0.049). mOS in the overall population was 62 months (CI 95% 35–74 months) (Figure 1a).

The PFS of first-line treatment, analyzing the population according to BMI status, was increased in high-BMI patients, with a 3-year PFS of 47% (mPFS 30 months, CI 95% 15–41 months) compared with 19% (mPFS 12 months, CI 95% 4–48 months) of the low-BMI population (*p* = 0.019); mPFS in the overall population was 22 months (CI 95% 13–30, 3 years PFS 30.3%) (Figure 1b).

If the two patients with PS2 were removed from the analysis, first-line PFS continued to be superior in the high-BMI group (*p* = 0.032), while OS was not statistically significantly correlated with BMI status (*p* = 0.056).

**Figure 2:**
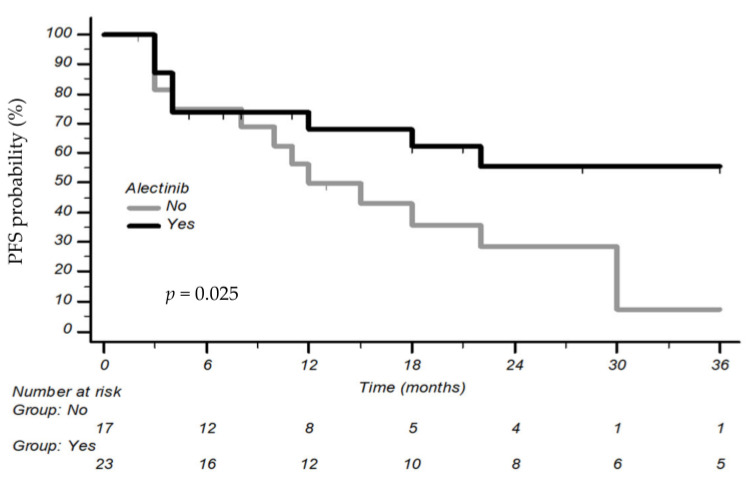
Progression-free survival of Alectinib treatment versus other anti-ALK treatments.

As expected, when we compared Alectinib treatment with other anti-*ALK* treatments, we discovered a higher 3-year PFS rate [55.6% vs. 7.1%, mPFS 41 months (CI 95% 12–41) vs. 12 months (CI 95% 4–48), respectively, *p* = 0.025] (Figure 2).

**Figure 3:**
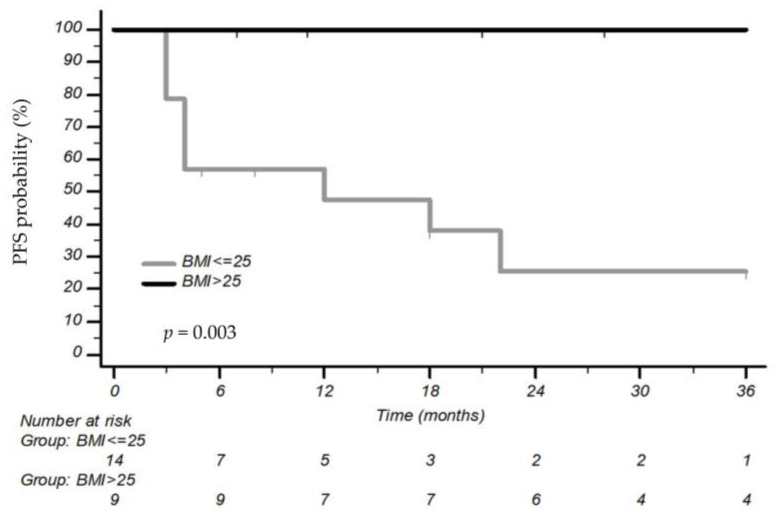
Progression-free survival according to body mass index (BMI) in patients treated with Alectinib as first-line treatment.

**Figure 4:**
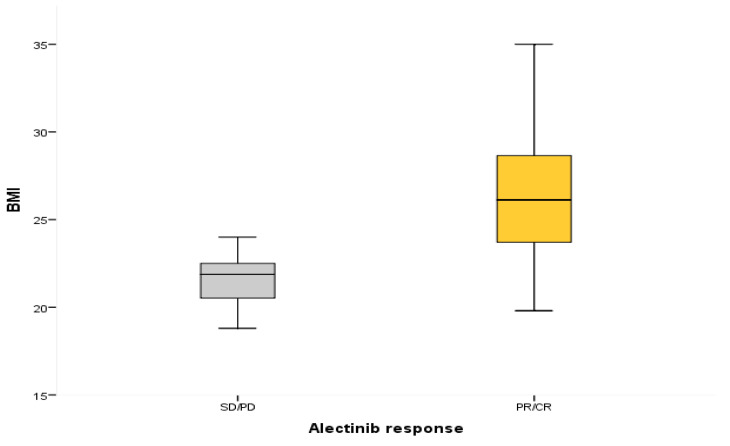
Overall response rate (ORR) measured by RECIST 1.1 Criteria according to body mass index (BMI) in patients treated with Alectinib as first-line treatment. CR = complete response; PD = progression disease; PR = partial response; SD = stable disease.

Interestingly, patients who received Alectinib as first-line treatment had a 3-year PFS rate of 100% if they were high-BMI versus only 25.4% in the low-BMI group (mPFS not estimated vs. 12 months, CI 95% 3–22 months, *p* = 0.03) (Figure 3). BMI positively correlated with ORR in the course of Alectinib as a first-line treatment (OR 1.71, CI 95%: 1.080–2.70, *p* = 0.022) (Figure 4). Notably, PFS was positively influenced by the interaction between BMI and Alectinib [HR 0.065, CI 95% 0.006–0.74, *p* = 0.028].

PFS and OS were not significantly influenced in univariate and multivariate analysis by clinical variables such as age at the metastatic stage, number of metastases, visceral metastasis, brain metastasis, and smoking status (Table 2).

At univariate analysis, high-BMI positively influenced PFS (HR 0.35, CI 95% 0.15–0.84, *p* = 0.019) and OS (HR 0.27, CI 95% 0.09–0.80, *p* = 0.020); at the same time, ECOG PS0 correlated with PFS (HR 0.22, CI 95% 0.07–0.65, *p* = 0.007) and OS (HR 0.21, CI 95% 0.06–0.71, *p* = 0.013).

Furthermore, at multivariate analysis, BMI and PS were independent factors at PFS (BMI high vs. low: HR 0.39, CI 95% 0.16–0.96, *p* = 0.042; PS0 vs. PS 1–2: HR 0.24, CI 95% 0.08–0.73, *p* = 0.012) and OS (BMI high vs. low: HR 0.18, CI 95% 0.05–0.61, *p* = 0.006; PS0 vs. PS1-2 HR 0.15, CI 95% 0.04–0.55, *p* = 0.014).

Treatment regimens different from Alectinib were associated with a shorter first-line PFS at univariate analysis (HR 2.35, CI 95% 1.05–5.25, *p* = 0.038) but not at multivariate analysis adjusted by PS and BMI (HR 2.01, CI 95% 0.89–4.5, *p* = 0.094). 

No significative difference between Crizotinib and Alectinib as first-line treatment were observed (HR 2.07, CI 95% 0.85–5.02, *p* = 0.10).

**Figure 5:**
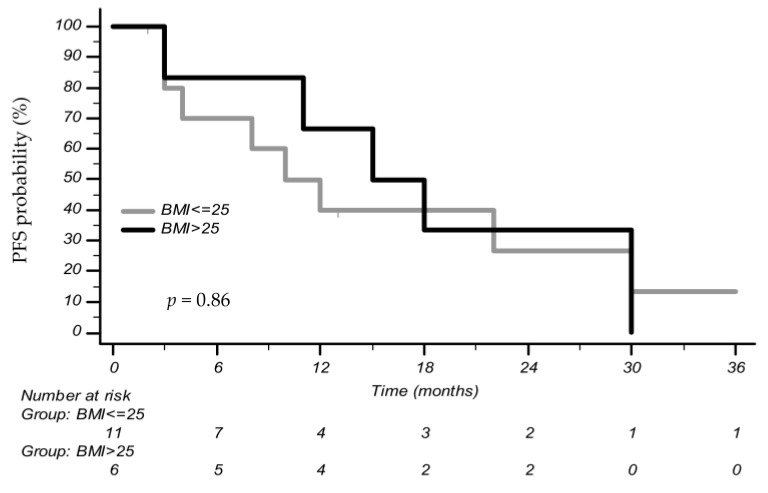
Progression-free survival (PFS) according to body mass index (BMI) in patients treated with other ALK inhibitors (ALKi) versus Alectinib.

In conclusion, we found some interesting clinical trends. Patients not treated with Alectinib who were grouped as high-BMI had a 3-year PFS of 0% compared to 13.3% of patients with a low BMI [mPFS 15 months (CI 95% 3–30) vs. 10 months (CI 95% 3–48), *p* = 0.86] (Figure 5). All patients who experienced a radiographic complete response (CR) (10 patients) in the course of *ALK* inhibitors as a first-line treatment (nine with Alectinib and one with Crizotinib) and as a second-line treatment (two with Alectinib) belong to the high-BMI category. All of them had PFS superior to 34 months at the time of last follow-up. Patients treated with metformin because of diabetes mellitus had a high BMI and a PFS superior to 34 months with Alectinib therapy. The presence of smoking history, collected in a large proportion of patients (48%), did not significatively influence ORR to first-line Alectinib treatment, although this was without statistical significance (OR 2.22, CI 95% 0.37–13.5, *p* = 0.38).

## 4. Discussion

To our knowledge, the present analysis is the first and only study that evaluates a possible positive correlation between BMI status and outcomes with *ALK* inhibitors treatment. Indeed, we divided the ALK-rearranged NSCLC population into two major groups, identifying a basal BMI of 25 mg/mq as a cut-off, as previously achieved in other studies [24,25,26]. 

Interestingly, overweight/obese patients had a better first-line PFS and OS and at the same time, if treated with first-line Alectinib, had a longer PFS compared to low/normal-BMI patients. Consequently, we believe that we have identified for the first time that BMI status could be a clinical predictive biomarker with Alectinib therapy.

However, the biological basis of the association is just beginning to be understood. ALK inhibitors were demonstrated to drastically increase lung cancer survival, but there is a lack of clinical and molecular prognostic and predictive biomarkers [28].

Zhou et al. observed that the BMI decreased the risk of lung adenocarcinoma after adjustment of smoking behaviors and increased the risk of small-cell lung cancer; it also increased the risk of lung squamous cell carcinoma, but this effect was mediated by smoking [29]. All NSCLC tissues evaluated in our study were *ALK*-rearranged adenocarcinoma, and only a few patients were current smokers, possibly explaining the beneficial effect of a higher BMI.

Immunological and nutritional markers may be useful in predicting the outcome of Alectinib as a first-line treatment. These data were evaluated at baseline and 3 weeks after the introduction of Alectinib in a retrospective study, and a longer duration of PFS was significantly associated with the baseline platelet-to-lymphocyte ratio, systemic immunoinflammatory index, and prognostic nutritional index [28].

In clinical lung cancer trials, patients have a high incidence of comorbidities, including diabetes mellitus (DM), considered an important component of the metabolic syndrome, and a possible risk factor for lung cancer. Nevertheless, the relationship between the two diseases remains unclear. Wang et al. showed that patients with DM had increased PFS and OS compared with those without, with an mPFS of 12 vs. 6 months and an mOS of 37 vs. 12 months, respectively [30,31]. 

Metformin, one of the most useful blood glucose-lowering drugs, improved survival in EGFR lung cancer and could be used to overcome HGF resistance to Alectinib. Indeed, in preclinical models, Metformin yielded the disruption of the MET-Gab1 complex and the inhibition of the phosphorylation of Gab1, a key downstream effector of the HGF/MET complex, and reversed Crizotinib resistance through the inhibition of IGF-1R signaling [32,33].

Few patients in this study had DM, and only four received Metformin, but all of them had a longer survival and complete response with Alectinib. However, it could be that MS, constituted by high-BMI and other pathways not known, leads to a beneficial effect on *ALK* TKIs.

Several trials studied the possible correlation between lung cancer and BMI with controversial results.

High baseline BMI positively correlated with improved lung cancer survival in different stages in different trials, probably because of its role in functional reserve. Meanwhile, the mechanisms of cancer cachexia are not fully understood, and some evidence has suggested that systemic inflammation plays a central role. Many factors could contribute to the pathogenesis of advanced lung cancer-induced cachexia: anorexia, cytokines, and energy and metabolic abnormalities [34,35,36]. 

Much evidence suggests a certain positive correlation between high-BMI lung cancer patients with immunotherapy but also with surgery, radiotherapy, and some types of chemotherapy. Curiously, prognosis in patients treated with an association of chemotherapy and immunotherapy as first-line treatment, or in the course of the antiangiogenetic agent, was not influenced by BMI. Also, obesity and excess adiposity seem to correlate with poorer survival among patients with NSCLC receiving platinum-based chemotherapy [37,38,39,40]. 

As previously mentioned, controversial results emerged in the *EGFR* NSCLC population. In the first-line setting, *EGFR* TKIs yielded better survival outcomes in overweight patients in two different trials; in the second-line, obesity negatively influenced outcomes. This analysis has never been conducted in *ALK*-rearranged patients treated with ALK TKIs [25,26,27,41].

On the other hand, BMI could not fully account for body composition because it could not explicate the differences in the ratio of muscle and diverse types of adiposities and the possible influence of endogenous (e.g., sex and race) and exogenous factors (e.g., tobacco consumption) with body habitus [42]. 

A large, pooled analysis investigated the impact of race, smoking, and sex on the relationship between BMI and OS; female ever-smoker white patients were the most heavily associated with better outcomes with a normal BMI, but this was not the case for underweight/obese patients. Moreover, BMI in Asian patients and never-smokers was not significantly associated with OS. These clinical characteristics are generally typical of the *ALK*-rearranged population [42].

Lung cancers are known to be aggressive, and patients with advanced disease usually have poorer PS and experience significant weight loss at the time of diagnosis [43]. Notably, in this study, BMI status influenced outcomes independently from basal PS status. 

Unfortunately, this study shows some limitations within which these results need to be interpreted. First of all, the retrospective design of the study and the collection of a small number of patients in a single institution may have prevented us from finding a significant correlation between *ALK* inhibitor outcomes and BMI in the NSCLC population. Moreover, no evaluation of nutritional status has been conducted because data could not be collected uniformly in all the populations, and there is a possible bias connected to nutritional evaluation. Consequently, our study is a future starting point for the evaluation of BMI as a predictive biomarker in the *ALK* NSCLC population, but results should be interpreted with caution. 

Considering the actual challenge of Brigatinib, Alectinib, and Lorlatinib as the best *ALK* inhibitor according to recent phase III trials results, we believe that it could be useful to identify any possible factor that could predict survival with Alectinib, directing the physicians’ choices of the best-fitting ALK inhibitor for each patient. 

Recently, a prospective trial analyzed the influence of Alectinib on body change composition; they demonstrated that Alectinib can cause a significant increase in sarcopenic abdominal obesity soon after initiation. This can lead to many serious metabolic, physical, and mental disturbances in long-term survivors [44].

Based on our results, we suggest that the next step in the development of an effective ALK treatment might be to evaluate the relationship between body size variables (evaluated with impedancemetry tests) and efficacy in the course of *ALK* inhibitors in NSCLC patients, also considering the influence of weight gain as a common side effect of *ALK* TKIs.

## 5. Conclusions

ALK inhibitors represent the gold standard of *ALK*-rearranged NSCLC treatments, providing significant benefits. We propose this study as a future starting point for a more substantial analysis of the correlation between response to *ALK* inhibitors and BMI status, also exploiting possible correlation with metabolic syndrome and molecular subtypes of lung cancer. We found a longer OS and PFS during *ALK* treatment in a small cohort of lung cancer patients with baseline high-BMI, particularly if treated with Alectinib as a first-line treatment. As shown by the multivariate analysis for first-line PFS and OS, the positive effect of BMI was not influenced by basal ECOG PS. However, these data should be further investigated to identify possible clinical factors to personalize the best *ALK* inhibitors treatment sequence.

## Figures and Tables

**Table 1:** Clinicopathological characteristics of patients. BMI = body mass index; CNS = central nervous system; DM2 = diabetes mellitus type 2; PS = performance status.

Variable	Level	Overall (N = 40)
Sex	Female	25 (62.5%)
	Male	15 (37.5%)
PS	0	13 (32.5%)
	1	25 (62.5%)
	2	2 (5%)
BMI	Low	25 (62.5%)
	High	15 (37.5%)
Smoking status	Non-smoker	21 (52.5%)
	Smoker	7 (17.5%)
	Former smoker	12 (30%)
DM2	Yes	7 (17.5%)
	No	33 (82.5%)
Metformin	Yes	4 (10%)
	No	36 (90%)
Steroids	Yes	21 (52.5%)
	No	19 (47.5%)
Antiepileptic drugs	Yes	16 (40%)
	No	24 (60%)
Visceral metastasis	Yes	32 (80%)
	No	8 (20%)
CNS metastasis	Yes	20 (50%)
	No	20 (50%)
Number of metastases	≤3	13 (32.5%)
	>3	27 (67.5%)
I line	Alectinib	23
	Crizotinib	10
	Brigatinib	2
	Chemotherapy	3
	Immunotherapy	2
II line	No treatment	23
	Alectinib	9
	Crizotinib	2
	Brigatinib	1
	Lorlatinib	2
	Ceritinib	1
	Chemotherapy	2
III line	No treatment	33
	Alectinib	2
	Lorlatinib	4
	Chemotherapy	1

**Table 2:** Univariate (**a**) and multivariate analysis (**b**) of PFS and OS. Statistically significant values are colored in orange and blue. ALKi = *ALK* inhibitors; BMI = body mass index; CNS = central nervous system; HR = hazard ratio; OS = overall survival; PFS = progression free survival; PS = performance status.

Variables	PFS Univariate Analysis	OS Univariate Analysis
(**a**)		
Smoking status	HR 1.85 (CI 95% 0.81–4.22) *p* = 0.15	HR 1.36 (CI 95% 0.49–3.81) *p* = 0.56
Yes vs. No		
Age at metastatic stage	HR 1.06 (CI 95% 0.98–1.03) *p* = 0.74	HR 1.03 (CI 95% 0.99–1.07) *p* = 0.22
Number of metastases	HR 1.45 (CI 95% 0.58–3.67) *p* = 0.43	HR 1.02 (CI 95% 0.36–2.91) *p* = 0.96
Visceral metastasis	HR 1.85 (CI 95% 0.54–6.26) *p* = 0.33	HR 3.68 (CI 95% 0.49–27.9) *p* = 0.21
Yes vs. No		
CNS metastasis	HR 1.61 (CI 95% 0.72–3.58) *p* = 0.24	HR 1.08 (CI 95% 0.41–2.85) *p* = 0.87
Yes vs. No		
PS0 vs. PS1/2	HR 0.22 (CI 95% 0.07–0.65) *p* = 0.007	HR 0.21 (CI 95% 0.06–0.71) *p* = 0.013
BMI high vs. BMI low	HR 0.35 (CI 95% 0.15–0.84) *p* = 0.019	HR 0.27 (CI 95% 0.09–0.80) *p* = 0.020
First-line ALKi	HR 2.35 (CI 95% 1.05–5.25) *p* = 0.038	-
Others vs. Alectinib		
(**b**)		
PS0 vs. PS1/2	HR 0.24 (CI 95% 0.08–0.73) *p* = 0.012	HR 0.15 (CI 95% 0.04–0.55) *p* = 0.014
BMI high vs. BMI low	HR 0.39 (CI 95% 0.16–0.96) *p* = 0.042	HR 0.18 (CI 95% 0.05–0.61) *p* = 0.006
First-line ALKi	HR 2.01 (CI 95% 0.89–4.5) *p* = 0.094	-
Others vs. Alectinib		

## Data Availability

The data that support the findings of this study are available from the corresponding authors, M.S. and A.J.G., upon reasonable request.
